# Multichannel Investigation of Interoception: Sensitivity Is Not a Generalizable Feature

**DOI:** 10.3389/fnhum.2018.00223

**Published:** 2018-06-01

**Authors:** Eszter Ferentzi, Tamás Bogdány, Zsuzsanna Szabolcs, Barbara Csala, Áron Horváth, Ferenc Köteles

**Affiliations:** ^1^Doctoral School of Psychology, ELTE Eötvös Loránd University, Budapest, Hungary; ^2^Institute of Health Promotion and Sport Sciences, ELTE Eötvös Loránd University, Budapest, Hungary; ^3^Institute of Psychology, ELTE Eötvös Loránd University, Budapest, Hungary

**Keywords:** interoception, interoceptive sensitivity, heartbeat perception, pain, water load test, balance, bitter taste

## Abstract

**Objective**: The term interoception refers to the perception of bodily cues. In empirical studies, it is assessed using heartbeat detection or tracking tasks, often with the implicit assumption that cardioception reflects general interoceptive ability. Studies that applied a multichannel approach measured only a limited number of modalities. In the current study, six modalities were assessed to gain a deeper understanding of the relationship between the different sensory channels of interoception.

**Methods**: For 118 university students (53% male) gastric perception (water load test), heartbeat perception (Schandry task), proprioception (elbow joint), ischemic pain (tourniquet technique), balancing ability (one leg stand), and perception of bitter taste were measured. Pair-wise correlation analysis and exploratory factor analyses (principal component analysis (PCA) and maximum likelihood (ML) extraction with oblimin rotation) were then carried out with a three-factor solution to investigate the underlying associations.

**Results**: Correlation analysis only revealed significant associations between variables belonging to the same sensory modality (gastric perception, pain, bitter taste). Similarly, the three factors that consistently emerged in the factor analyses represented the three aforementioned modalities.

**Discussion**: Interoceptive sensitivity assessed by using one channel only cannot be generalized. Interoceptive modalities carrying crucial information for survival are not integrated with other channels.

## Introduction

Interoception, the perception of the state of the body (Ceunen et al., 2016) is a multimodal construct that includes several physiological channels. Beyond modality-specific information, integrated interoceptive information provides the sense of the physiological condition of the entire body (Craig, 2002, 2003a), the basis for subjective feeling states (Craig, 2002), and the sense of self (Damasio, 1999; Craig, 2015). Interoception originally only referred to visceroception (Ceunen et al., 2016), but recent neuroanatomical findings support the notion that a wide variety of bodily information becomes integrated at the level of the insula, contributing to the maintenance of homeostasis, and providing the subjective *“Gemeingefühl”* or common sensation, i.e., how we feel in our body (Craig, 2015). These findings are in line with a more inclusive conceptualization of interoception (Ceunen et al., 2016).

There are two distinct neural pathways of interoceptive information (Craig, 2015). One ascends from the skin, muscles and joints through the medial lemniscus or the analog cranial nerves to the thalamus and ends in the somatosensory cortex. It carries proprioceptive and mechanical (tactile) information. The other pathway relays the primary visceral information upward to another region of the thalamus using the spinothalamic tract; then it reaches the insula (Craig, 2009). This tract is responsible for a wide variety of sensations, such as pain, temperature, affective touch and visceral sensation (Craig, 2015). The information provided by these two pathways (along with the input from the gustatory system; Avery et al., 2015) is integrated at multiple levels, among which the medial and the anterior insular cortex play the primary role (Craig, 2015). As a consequence, the anterior part of the insular cortex can be activated by a variety of stimuli, such as taste, thirst, sensual touch, itch, sexual arousal, warmth and distension of the stomach or rectum (Craig, 2004, 2009). For example, the heightened activity in the right (non-dominant) anterior insular/opercular cortex, as measured with functional MRI, predicted the performance level in the heartbeat detection task (Critchley et al., 2004).

Despite the multiple integrations of the interoceptive information in the central nervous system, it is an open question whether we can talk about general interoceptive ability, and how this could be described and understood. It was proposed that great intra- and interindividual variability exist regarding the accuracy of different interoceptive channels (Vaitl, 1996). For example, someone with a good performance in cardiac perception is not necessarily sensitive to other bodily cues, such as signals originating in the gastrointestinal or respiratory system (Vaitl, 1996). Until recently, both the number of empirical studies investigating multiple interoceptive channels simultaneously, and the number of the investigated modalities have been limited.

Most of the empirical results support the supposition of Vaitl (1996), except two studies that compared the cardiac and the gastrointestinal system. An earlier study found a medium level correlation between the accuracy of detection of heartbeats and stomach contractions (Whitehead and Drescher, 1980). The same level of association between cardiac and gastric perception was demonstrated using a different methodology, i.e., the heartbeat tracking and water load test paradigms (Herbert et al., 2012). Another study investigated three channels and found significant moderate associations between the perception of skin conductance level (assessed by the sensation of dry vs. sweaty hands) and the two other measured variables, namely the perception of heart rate and respiratory resistance. However, the latter two did not correlate with each other (Steptoe and Noll, 1997). It is important to emphasize that the effect size of the aforementioned associations is in the moderate domain, explaining only 13%–25% of the total variance (Whitehead and Drescher, 1980; Steptoe and Noll, 1997; Herbert et al., 2012).

The majority of empirical studies did not find associations between the investigated modalities. A study encompassing heartbeat discrimination and respiratory resistance tasks applying signal detection theory found that neither perceptual accuracy nor response bias were related among the tasks (Harver et al., 1993). A recent study reported similar results (Garfinkel et al., 2016). A comparison between the threshold and tolerance for heat pain and the results of heartbeat tracking task found no association between the sensation of pain and cardiac activity (Werner et al., 2009). Our recent study investigated four interoceptive channels, namely heartbeat perception with the tracking task, pain threshold and tolerance in induced ischemic pain, bitterness sensitivity and balancing ability (Ferentzi et al., 2017). Again, no correlations were found between any of the investigated sensory channels. Another study that reported preliminary results revealed an association between cardiac and gastric accuracy, but no connection between these variables and respiratory sensitivity was found (Garfinkel et al., 2017). It is important to note, however, that the interpretation of the results of this study is problematic due to the small sample size and lack of methodological information.

Another way to define the general interoceptive ability is not to presume that the sensitivity and accuracy of different interoceptive channels are more or less similar in magnitude. Instead, the combination or integration of those interoceptive channels may provide some kind of general interoceptive ability. The fact that information originating in various interoceptive channels becomes more and more integrated in higher levels of central processing, and also the concept that this integration has a homeostatic function in Primates (i.e., the assessment of the general state of the body; Craig, 2015) support this assumption. Empirical testing of this hypothesis, however, is difficult, as the general feeling about the body is measurable only via self-report. Interoceptive awareness (Ceunen et al., 2013) or sensibility (Garfinkel and Critchley, 2013; Garfinkel et al., 2015), defined as self-reported interoceptive ability, might be biased in different ways. Questionnaires, usually assessing a variety of interoceptive channels, are meant to measure the general perceived interoceptive ability, but they are influenced heavily by memory and subjective interpretation. Additionally, the interoceptive ability level assessed with questionnaires is not associated with the level investigated with behavioral tasks (Garfinkel et al., 2015).

The available information concerning the relationships of the accuracy of various interoceptive channels is scarce and inconclusive. Although the contemporary broad conceptualization of interoception includes the lemniscus medialis pathway (proprioceptive and tactile information), the integration of these modalities with those transmitted by the spinothalamic tract has not been investigated systematically to date. Additionally, most studies with multichannel approach measured only a limited number of modalities.

The aim of the present study is to investigate whether information obtained from a single interoceptive modality can be generalized to other modalities. According to our hypothesis, there will be no considerable associations among various interoceptive channels, except of the association between heartbeat perception and gastric sensitivity.

## Materials and Methods

### Participants

For factor analysis, the primary statistical method applied in the current study, no formal* a priori* sample size calculation can be conducted. Taken into consideration the complexity of the study and the expected high drop-out rate, our pre-determined goal was achieving a variables-to-factors ratio of 10 (Everitt, 1975) after the exclusion of participants with missing data from more than two sensory modalities.

Undergraduate university students (*N* = 142, 54% male; age: 21.93 ± 3.582) participated in the study. Individuals with missing data for more than two tasks out of the six were excluded (*N* = 24). In the final sample (*N* = 118, 53% male; age: 21.72 ± 3.007), data were missing for 11 participants for the heartbeat perception task, 29 participants for the water load task, 20 participants for the bitterness task, 17 participants for the pain task, five participants for the proprioceptive tasks, and eight individuals for the balancing task.

The study was carried out in accordance with the Declaration of Helsinki. The protocol was approved by the Research Ethics Committee of the Faculty of Education and Psychology, ELTE Eötvös Loránd University, Budapest, Hungary. All subjects signed an informed consent form before the measurements.

### Sensory Measurements

We used six different sensory measurements in the present study. Compared to our previous investigation (Ferentzi et al., 2017), we implied two main modifications. First, the proprioceptive modality was also included. Second, we investigated one more “classic” visceroceptive channel, namely gastric perception.

#### Heartbeat Perception Task

To assess the ability to perceive heartbeats, the slightly modified version of the tracking task of Schandry was conducted (Schandry, 1981), just like in our previous study (Ferentzi et al., 2017). Participants counted their heartbeats silently in a seated position through three time periods (30 s 45 s and 100 s in random order; after a 15 s long trial). Accuracy of perception was calculated for each trial using the following formula: 1 − |(recorded heartbeats − counted heartbeats)/recorded heartbeats|; which was followed by the calculation of the mean score. Higher scores refer to higher levels of accuracy. Participants were instructed to breathe regularly; they were not allowed to take their pulse or use other techniques that could help counting. They were encouraged to only count those heartbeats they were sure about, but also instructed to take into account weak sensations.

#### Water Load Test

Gastric perception ability was assessed by a non-invasive method, a modified version of the water load test (Boeckxstaens et al., 2001). The duration of the task was 5 min; participants drank the same amount of water (adjusted to their height in cm; e.g., 175 maximum likelihood (ML) with a height of 175 cm) in every minute. After each dose, they rated the sensation of gastric fullness and gastric unpleasantness, using a 10 cm long visual analog scale. The difference between the fifth and the first rating was calculated for both scales. Higher scores indicate higher sensitivity to gastric distension.

#### Bitterness Sensitivity

Sensitivity to taste was measured using an extract that was prepared using a herbal *(Centaurii herba)* by steeping 1 g of the dry plant in 1 l hot water for 5 min. Participants were asked to taste the liquid and to rate how bitter (bitter intensity) and how unpleasant (bitter unpleasantness) it was, using a 10 cm long visual analog scale; higher values indicated higher levels of subjective perceptions. The same method was used in our previous study (Ferentzi et al., 2017).

#### Pain

Pain threshold and tolerance were assessed using a modified version of the tourniquet technique (Amanzio and Benedetti, 1999), as we used in our previous study (Ferentzi et al., 2017). Subjects were in a lying position with their forearm extended vertically, while venous blood was drained from the arm with an Esmarch bandage; then sphygmomanometer placed around the upper arm was inflated to 300 mm Hg. Participants were asked to squeeze a hand grip 12 times; each squeeze lasted 2 s with 2 s rest in between. The resistance of the exerciser was set to 10 kg. The time of pain threshold (i.e., when the sensation was first described as pain) and pain tolerance (i.e., when the pain became unbearable) were registered in seconds, starting the time measurement after the last squeeze.

#### Proprioceptive Task

The proprioceptive sensitivity of the elbow joint was investigated using the modified version of the device of Goble (2010). Participants were in a seated position with the elbow placed on a rotatable board at shoulder height, eyes closed. The first task was to reproduce the position of the same forearm by moving the elbow only (proprioception, one arm), while the second task was to replicate the position of the opposite forearm (proprioception, two arms). Ten trials were conducted per task, five per arm. The dominant and non-dominant arms were investigated in random order. Before each trial, the arm of the participants was always fully stretched. Positions to replicate were randomly presented between 30 degrees and 150 degrees. Proprioceptive sensitivity was calculated by the mean of the difference between the target and reproduced position (in degree), using the results of the dominant arm only. Higher scores refer to lower levels of accuracy in proprioception.

#### Balancing

Processing of vestibular information was measured using a balancing task. Participants were asked to stand on one leg with closed eyes. Balance ability was assessed with the average time length (s) of two trials of standing without step downs (max: 60 s). With open eyes, all participants were able to stand on one leg for a minute, which indicates that physical skills did not limit their balancing performance. Higher scores refer to better balancing ability.

### Protocol

All the participants were assessed in a 5-week time period. Balance and proprioceptive abilities were assessed at the same occasion (in random order), just like the sensitivity to bitter taste and the water load test (always bitter taste first, not to modify bitter sensitivity by the feeling of fullness). Apart from that, sensory measurements occurred in random order at four different appointments.

### Statistical Analysis

Data were analyzed with the SPSS v21s software. Correlations among variables were estimated using Spearman correlation. Because of the large number (45) of correlation analyses, the accepted level of significance was set to 0.001 (Bonferroni correction). As data was appropriate for exploratory factor analysis (KMO = 0.389; Bartlett’s test *p* < 0.001), principal component analysis (PCA) and factor analysis with ML extraction (both with Oblimin rotation) were chosen to explore combined associations of variables. The problem of missing data was addressed by using a matrix of expectation maximization correlations as input for the factor analyses (Weaver and Maxwell, 2014).

## Results

Descriptive statistics and Spearman correlations are presented in Table 1. Only three significant (*p* < 0.001) correlations were found, consistently between the two indicators of same interoceptive channel, i.e: (1) pain (*ρ* = 0.51); (2) sensitivity to bitter taste (*ρ* = 0.80); and (3) gastric sensitivity (*ρ* = 0.48). All other correlations were non-significant at the adjusted level of *p* (0.001), and the absolute value of the majority of correlation coefficients (which is considered an effect size indicator) was in the 0.0–0.1 domain.

**Table 1:** Descriptive statistics (M ± SD) of and Spearman correlations between the assessed variables.

	Pain threshold (s)	Pain tolerance (s)	Balance (s)	Bitter intensity	Bitter unpleasantness	Gastric fullness	Gastric discomfort	Heartbeat tracking	Proprioception, one arm	Proprioception, two arms
Pain threshold (s)	173.90 ± 246.154	0.51*	0.07	−0.02	0.01	0.01	−0.34	0.07	−0.03	−0.10
Tolerance (s)		577.45 ± 379.227	−0.05	−0.07	−0.03	−0.08	−0.18	0.06	−0.02	0.10
Balance (s)			24.13 ± 18.612	−0.05	0.00	−0.01	−0.13	−0.01	−0.06	−0.10
Bitter intensity				44.86 ± 23.653	0.80*	−0.13	0.10	0.05	−0.20	0.01
Bitter unpleasantness					37.31 ± 26.084	−0.16	0.10	0.05	−0.32	0.05
Gastric fullness						34.25 ± 22.974	0.48*	−0.11	−0.05	−0.01
Gastric discomfort							27.84 ± 26.909	−0.26	−0.12	−0.01
Heartbeat tracking								0.54 ± 0.272	−0.00	0.06
Proprioception, one arm									5.63 ± 2.686	−0.03
Proprioception, two arms										6.30 ± 3.223

Note: *p < 0.001 (Bonferroni corrected level of significance).

Concerning the exploratory factor analysis, although communalities of three variables (the two proprioceptive variables and heartbeat tracking) were rather low (<0.4), we decided to keep them. According to the results of an exploratory PCA, the first four components had an eigenvalue larger than 1 (2.027; 1.743; 1.562; 1.092). Considering the shape of the scree plot, a three-factor solution appeared to be the best option (the first three factors explained 53.3% of the total variance). Thus, two other analyses with three factors using PCA and ML extraction method with oblimin rotation were conducted. Rotated structure matrices from the PCA and ML extraction are presented in Tables 2 and 3. Correlations among extracted components were negligible (≤0.2) in all cases. No generalized underlying factor (i.e., a dimension with a disproportionally high eigenvalue and substantial loadings of various sensory modalities) was revealed. Indicators related to bitter taste, distension of the stomach, and pain clearly loaded on different factors in both analyses. In the output of the PCA (Table 2), the one-arm proprioception task was reversely connected to the bitterness factor (Factor 1), while heartbeat tracking negatively loaded on the gastric factor (Factor 2). The two-arm proprioception task and balance did not load on any factor. Concerning the results of the ML extraction (Table 3), the one-arm proprioception task was reversely connected to the bitterness factor (Factor 3) again. The two-arm proprioception task, heartbeat tracking, and balance showed no considerable loading on any factor.

**Table 2:** Variables’ loadings on the three factors yielded by principal component analysis (PCA) with Oblimin rotation (values larger than 0.3 are marked with bold).

	Factor 1	Factor 2	Factor 3
Gastric fullness	−0.090	**0.856**	0.162
Gastric discomfort	0.179	**0.889**	−0.094
Heartbeat tracking	0.154	**−0.334**	0.161
Proprioception, one arm	**−0.539**	−0.164	−0.238
Proprioception, two arms	0.085	−0.033	0.067
Pain threshold	0.110	−0.041	**0.840**
Pain tolerance	0.071	−0.147	**0.841**
Balance	−0.121	−0.153	−0.231
Bitter intensity	**0.893**	−0.055	0.014
Bitter unpleasantness	**0.910**	−0.098	0.044

**Table 3:** Variables’ loadings on the three factors yielded by maximum likelihood (ML) extraction with Oblimin rotation (values larger than 0.3 are marked with bold).

	Factor 1	Factor 2	Factor 3
Gastric fullness	0.139	**0.687**	−0.067
Gastric discomfort	−0.151	**0.960**	0.225
Heartbeat tracking	0.011	−0.242	0.062
Proprioception, one arm	−0.035	−0.087	**−0.327**
Proprioception, two arms	−0.085	−0.015	0.029
Pain threshold	**0.990**	0.056	0.103
Pain tolerance	**0.521**	−0.033	0.006
Balance	−0.027	−0.098	−0.048
Bitter intensity	−0.111	−0.021	**0.862**
Bitter unpleasantness	−0.107	−0.082	**0.917**

## Discussion

In an experimental study investigating the perception of a total of 10 variables belonging to six sensory channels (gastric perception, heartbeat perception, proprioception, pain, balance and bitter taste), no between-channel connections and a general factor underlying interoceptive sensitivity were found. In the correlation and factor analyses, the different aspects of the same channel (i.e., fullness and discomfort of the stomach, intensity and unpleasantness of the bitter taste, and pain threshold and tolerance) consistently loaded on the same factor, supporting the notion, that modalities themselves provide congruent and strategic information. An exception was the proprioceptive test, where the two versions loaded on different factors. The two tasks might require different abilities: while utilization of short-term memory is needed for the one arm version, communication between the two hemispheres is required for the task conducted with two arms (Goble, 2010). This is the first study that investigates the relation of several distinct interoceptive channels including “classic” visceroceptive modalities as well as channels that are related to proprioception or activated by possibly dangerous external stimuli.

The findings of previous studies comparing gastric sensitivity and heartbeat perception abilities (Whitehead and Drescher, 1980; Herbert et al., 2012) have not been replicated; on the contrary, PCA revealed a negative connection between the two channels. However, it is noteworthy that the methodology of both past studies differed from that of our study. Whitehead and Drescher (1980) used the perfused catheter method to assess stomach contractions, and the subjects had to decide whether the contractions coincided with an external light signal. Heartbeat detection accuracy was measured using a comparable method (Whitehead-paradigm), based on signal detection theory. The study of Herbert et al. (2012) interpreted the amount of consumed water as the measure of gastric sensitivity, as participants were instructed to drink until they reach the point of perceived fullness; while in the recent study the stimulation (i.e., the amount of water) was kept constant. Heartbeat perception ability was assessed using the Schandry mental tracking paradigm in the study of Herbert and colleagues as well as in the present study. In our research, however, perceived fullness and unpleasantness of the stomach might have been higher as a consequence of the forced drinking paradigm, particularly for those with higher sensitivity to negative sensations originating in the visceral region. A tendency to negative evaluation (i.e., negative affect) was reversely connected with heartbeat perception in past studies, and was explained as a negative cognitive bias that interferes with perceptual processes (Barsky et al., 1995; Aronson et al., 2001; Aamland et al., 2012; van den Bergh et al., 2004).

For the other non-expected negative correlation (i.e., the one-arm proprioceptive task was reversely connected with the bitterness factor in both analyses), we could not find any satisfying explanation. A distinct feature of the former task is that it requires short-term memory and cognitive effort, which can be negatively influenced by automatic evaluative processes. From this aspect, however, a negative connection with the pain factor would also be reasonable, both anatomically (the ischemic pain test was also conducted on the arm) and conceptually. To gain a better understanding of this finding, replication of the connection and an experimental study dedicated to the issue would be necessary.

### General Interoceptive Ability

Overall, our findings strongly support the idea that interoceptive accuracy assessed with a single modality cannot be generalized across various channels. It is particularly striking that the widely used heartbeat tracking task showed no substantial connection with any of the interoceptive channels. In fact, even various aspects of cardioception (heartbeat detection, pulse rate perception, perception of arrhythmias) show no significant associations with each other (Barsky et al., 1993). To prevent confusion, our suggestion is to use the expression of “heartbeat perception accuracy” instead of the misleading “interoceptive accuracy,” if only heartbeat perception is assessed. It is also important to note that the conclusion that interoceptive modalities are not independent from each other was drawn solely from the medium level association between cardiac and gastric perception. However, the connections between gastric sensation and other modalities were not investigated to date.

There are two possible conceptualizations of general interoceptive ability. One option is that it is manifested at the same level in every channel, i.e., the accuracy of different interoceptive modalities ought to be more or less the same, or at least correlate strongly for each individual. Findings of the present study, as well as those of previous studies with a medium effect size (Whitehead and Drescher, 1980; Steptoe and Noll, 1997; Herbert et al., 2012) do not support this possibility. Generalization across modalities or inferring from one modality to another does not appear to be a good practice in interoception related research or treatment. The other option is that general interoceptive ability cannot be measured by focusing on individual channels, because it represents the integration or combination of the accuracy levels of all the possible interoceptive modalities; thus, there is no strong association among modalities. This assumption is not contradictory to the findings available so far. Although empirical studies show that interoceptive information is integrated at various levels of the nervous system, it is an open question, how this integration is accessible by behavioral measurement methods (i.e., interoceptive tasks).

A recent model proposed by Smith and Lane (2015) describes three stages of the perception of body and emotions: (1) discrete body features; (2) whole-body patterns; and (3) emotion concepts. By referring to other authors (Jackendoff, 1987; Prinz, 2004) they argue that stage 2 processes correspond to the phenomenological differences in the individual experiences, which are represented as a coherent whole-body pattern. Consequently, we do not typically experience discrete bodily cues that are linked to specific organs.

It is often assumed that interoceptive accuracy (Ceunen et al., 2013; Garfinkel et al., 2015) or sensitivity, represent an objective measure (Critchley et al., 2013). The behavioral measurement, however, can also be biased by autonomic evaluation and appraisal (Smith and Lane, 2015). In accordance with this view, a previous study found that the perceived unpleasantness of bitter taste and pain threshold/tolerance were related to somatosensory amplification, the tendency to experience the bodily sensations as intense, noxious and disturbing; while heartbeat tracking and balancing ability, and the intensity of bitter taste were not (Ferentzi et al., 2017). This result indicates that the evaluation of the interoceptive signals that have a threatening quality and can be regarded as “homeostatic emotions” (Craig, 2003b), might be more likely to evoke responses, than the evaluation of the information belonging to other sensory channels. In other words, ascending sensory information is subject to low-level evaluation. As “discrete body features” are integrated at the level of the mid and anterior insula to the “whole-body pattern” (Smith and Lane, 2015), a behavioral or verbal report of these sensations is necessarily preceded by low level evaluative processes. Due to these early automatic processes, the objective comparison of different introceptive channels is problematic.

Thus, the functions of the interoceptive channels and their relation to other psychological factors have a crucial role in the interpretation of our results. The three channels that loaded consistently on different factors (pain, bitterness and gastric perception) in our findings represent three distinct subjective sensations. Even if we (based on intuition) presume the existence of a general interoceptive sensitivity level, it might be the case that these modalities have different significance and function than the general interoceptive level. The information provided by these channels is important for the organism in its own right. Therefore, they do not seem to contribute to the “common sensation” but have their distinct and clearly recognizable representations. This is in line with the presumption that different interoceptive channels are not equally relevant from the viewpoint of survival (Ferentzi et al., 2017).

### Single and Multichannel Approaches

In some specific cases, a single interoceptive modality is significant on its own. Thus, the investigation of this modality might be warranted. For example, increased heart rate, an indicator of higher arousal, is a frequently described characteristic of anxiety disorders. Therefore, heartbeat perception tasks might be a relevant tool to assess this specific interoceptive sensitivity (Willem van der Does et al., 2000; Domschke et al., 2010). Similarly, some variations of the water load test might be a helpful paradigm to explore the background of gastric disorders, e.g., functional dyspepsia (Koch et al., 2000; Boeckxstaens et al., 2001; Mimidis, 2007).

There are cases, on the other hand, where the investigation of a single channel does not appear an appropriate practice. For example, although meditation is assumed to improve the sensation of bodily signals, several studies using heartbeat perception task did not show differences between meditators and non- meditators (Nielsen and Kaszniak, 2006; Khalsa et al., 2008; Melloni et al., 2013; Otten et al., 2015), while one study using respiratory task led to mixed results (Daubenmier et al., 2013).

There are multichannel paradigms of interoception that represent a different approach. A study investigated the ability of healthy young adults to adjust their cardiovascular parameters during bicycle ergometer exercise to a previously experienced level (Kollenbaum et al., 1996). Out of the measured variables (heart rate and blood pressure) only heart rate was reproduced with high consistency by participants, which supports the idea that interoceptive accuracy cannot be generalized across channels.

Physiological or neurological dysfunctions and the chronic mal-functionality of an interoceptive channel might represent a good opportunity to understand the interoceptive system better. A study introducing a patient with external cardiac assist supports the existence of different neural pathways of heartbeat perception (Couto et al., 2014). Another study investigated two patients with focal brain lesions, using heartbeat perception task, and taste, smell and thermal pain stimulation, and argued for distinct neurological background of the different interoceptive channels (Couto et al., 2015). Connections between channels are also described. For example, higher olfactory thresholds predicted higher interoceptive accuracy scores, however the duration of the disease of people with olfactory dysfunction indicated reduced heartbeat tracking abilities (Krajnik et al., 2015). Because of the close link between heartbeat perception accuracy and affect (Wiens et al., 2000; Pollatos et al., 2007), one has to be careful with the interpretation of findings on the connection between interoceptive accuracy and chronic mal-functioning. The existence of a third underlying variable might represent the best explanation for the seemingly direct connection. Although functional motor disorder is associated with lower heartbeat perception scores, reduced interoceptive accuracy also predicted depressive symptoms and self-objectification (Ricciardi et al., 2016).

### Limitations and Future Research

The present study is not without limitations. First, conceptually distinct approaches were used in the measurements. One type of the assessments applied external stimuli (i.e., pain, bitter liquid, water) to induce subjective ratings, while the perception of the more or less natural operation of the given sensory channel was measured by the assessment of heartbeat tracking and balance. Accordingly, the rating of the sensory channels was also different: in the case of pain, bitter liquid and water, the tasks measured the sensitivity to a standardized stimulation; while in the case of heartbeat tracking and balance, the tasks required internal focus without an additional stimulus. The proprioceptive task represents another approach that involves detection and active reproduction. Moreover, as automatic evaluation takes place at lower levels of central processing on ascending information, it is not easy to draw a line between the measures of interoceptive accuracy, subjective sensation and its subjective evaluation, especially as the later might be easily related to emotional states (Smith and Lane, 2015). From a theoretical point of view, these conceptual differences in the assessment of interoceptive accuracy make the direct comparison of different channels difficult, which might result in a decrease in the estimated strengths of associations. Concerning the statistical analysis, missing values were handled with pair-wise exclusion that might have impacted the results.

Other methodological issues might have also influenced the results. For example, heartbeat perception accuracy values obtained by different paradigms often show no or weak association only (Schulz et al., 2013; Ring et al., 2015). Similarly, the measurement of proprioception has several different paradigms (Han et al., 2016), and other proprioceptive tasks might relate to the other interoceptive channels differently. For example, the perception of the joints of the legs might be more connected with balance ability. The measurement of the gastric sensitivity also has different approaches, with the different types of water load or drinking tests (Mimidis, 2007) representing only a specific subtype. These conceptual and methodological differences should be taken into consideration in future multimodal interoception studies.

## Conclusion

Interoceptive sensitivity assessed by using one sensory modality only cannot be generalized to other modalities. Interoceptive channels carrying crucial information for survival (e.g., pain, bitterness and gastric perception) are not integrated with the other investigated channels.

## Author Contributions

EF and FK contributed to the conception and design of the study. ÁH, BC, EF, ZS and TB all contributed to the assessment and processing of data. FK performed the statistical analysis. EF wrote the first draft of the manuscript. KF wrote sections of the manuscript. All contributing authors read and commented on the last version of the manuscript.

## Conflict of Interest Statement

The authors declare that the research was conducted in the absence of any commercial or financial relationships that could be construed as a potential conflict of interest.
